# Active Inferants: An Active Inference Framework for Ant Colony Behavior

**DOI:** 10.3389/fnbeh.2021.647732

**Published:** 2021-06-24

**Authors:** Daniel Ari Friedman, Alec Tschantz, Maxwell J. D. Ramstead, Karl Friston, Axel Constant

**Affiliations:** ^1^Department of Entomology and Nematology, University of California, Davis, Davis, CA, United States; ^2^Active Inference Lab, University of California, Davis, Davis, CA, United States; ^3^Sackler Centre for Consciousness Science, University of Sussex, Brighton, United Kingdom; ^4^Department of Informatics, University of Sussex, Brighton, United Kingdom; ^5^Division of Social and Transcultural Psychiatry, Department of Psychiatry, McGill University, Montreal, QC, Canada; ^6^Culture, Mind, and Brain Program, McGill University, Montreal, QC, Canada; ^7^Wellcome Centre for Human Neuroimaging, University College London, London, United Kingdom; ^8^Spatial Web Foundation, Los Angeles, CA, United States; ^9^Theory and Method in Biosciences, The University of Sydney, Sydney, NSW, Australia

**Keywords:** ants, foraging, active inference, behavioral modeling, collective behavior, T-maze, eco-evo-devo, stigmergy

## Abstract

In this paper, we introduce an active inference model of ant colony foraging behavior, and implement the model in a series of *in silico* experiments. Active inference is a multiscale approach to behavioral modeling that is being applied across settings in theoretical biology and ethology. The ant colony is a classic case system in the function of distributed systems in terms of stigmergic decision-making and information sharing. Here we specify and simulate a Markov decision process (MDP) model for ant colony foraging. We investigate a well-known paradigm from laboratory ant colony behavioral experiments, the alternating T-maze paradigm, to illustrate the ability of the model to recover basic colony phenomena such as trail formation after food location discovery. We conclude by outlining how the active inference ant colony foraging behavioral model can be extended and situated within a nested multiscale framework and systems approaches to biology more generally.

## Introduction

### Bayesian Multiscale Modeling and Stigmergy in the Eusocial Insects

Systems biology starts from the recognition that biological function occurs in nested multiscale systems (i.e., the metabolic loop, the neural circuit, and the social group) (Noble et al., 2019). Biological phenomena are intrinsically multiscale because biological systems exist and influence events over several spatial and temporal scales, from microscale events within cells, to mesoscale phenomena, such as the adaptive behavior of single organisms, to macroscale phenomena like evolution by natural selection and ecological niche construction (Saunders and Voth, 2013; Roberts, 2014; Bellomo et al., 2015; Ramstead et al., 2019a). Eco-evo-devo is the new synthesis in biology that combines theories, methods, and insights from the study of ecology, evolution, and development (Abouheif et al., 2014; Sultan et al., 2017; Jablonka and Noble, 2019). Collective behavioral algorithms span scales of biological organization and explain the function and resilience of complex biological systems (Hills et al., 2015; Gordon, 2016; Feinerman and Korman, 2017). Studies of collective behavior within the Eco-evo-devo framework emphasize ecological context, subunit sensitivity to interactions, and emergent properties of groups (Bradbury and Vehrencamp, 2014; Friedman et al., 2020a; Gordon, 2020). The study of *multiscale optimization* in natural systems explores how interactions among system subunits results in system function, flexibility, and even optimality (Gordon, 2010; Friston et al., 2015; Rossi et al., 2018; Moses et al., 2019; Friedman et al., 2020a; Kuchling et al., 2020; Ramstead et al., 2020).

The eusocial insects [species beyond the point of no return of obligate colony living, such as ants, honey bees, termites, some wasps, etc. (Boomsma and Gawne, 2018; Friedman et al., 2020a)] have provided inspiration for developments in various fields, such as unconventional computing, autonomous robot swarms, disaster response strategies, and the regulation of physiology and behavior (Theraulaz and Bonabeau, 1999; Friedman et al., 2020a). In particular, collective foraging behavior, the set of processes by which resources are acquired by animal groups, are of special interest because it maps onto questions of computational tractability, economics, logistics, resource discovery, and information sharing amidst uncertainty (Wilson and Hölldobler, 1988; Lanan, 2014; Baddeley et al., 2019). Models of collective behavior can apply to groups of non-eusocial animals such as schools of fish. However in the case of the obligately eusocial animals, selection has acted over millions of years to shape their collective decision-making to be truly organismal (Wheeler, 1911), rather than based upon e.g., population-type game theory mechanisms and dynamics. Colony foraging processes are regulated via the complex interplay of ambient environmental conditions, interactions among nestmates, and nestmate variation in tissue-specific physiological variables (Abouheif et al., 2014; Warner et al., 2019; Friedman et al., 2020a,b). Collective foraging strategies in ants are variable across species, reflecting generalist or specialist approaches that exploit statistical regularities of various ecosystem niches (Lanan, 2014; Gordon, 2016, 2019).

While in many situations the regulation of ant colony foraging activity takes place through tactile interactions among nestmates (Greene et al., 2013; Razin et al., 2013), of particular interest for the present paper is the case of stigmergic regulation of colony foraging. *Stigmergy* is the phenomenon whereby accumulated traces left in the environment by agents are used to direct the behavior of their conspecifics (Heylighen, 2016). In ecological settings, pheromones produced by the insects are perceived in mixtures, in combination with other biotic and abiotic scents (Attygalle and Morgan, 1985). However it is also the case that single components of trail pheromones in isolation are able to induce nuanced behaviors such as attraction, repulsion, or initiation of action sequences. Sensitivity to trail pheromones and other cues are modulated by neurophysiological differences among nestmates and colonies (Mizunami et al., 2010; Muscedere et al., 2012; Rittschof et al., 2019). The evolution of stigmergic processes can be non-linear, as small changes in ecological context or to nestmate sensitivity can result in novel colony-level outcomes (Invernizzi and Ruxton, 2019). How colonies are able to use stigmergic regulation to generate adaptive collective behavior amidst uncertainty is a fundamental motivator of this work.

Recent work has framed the ant colony as a “Bayesian superorganism,” in that the colony engages in a form of collective decision-making via a stochastic sampling of environmental gradients by nestmates that can be modeled as a form of Bayesian inference (Hunt et al., 2016, 2020a,b; Baddeley et al., 2019). This colony-level behavior is Bayesian because colony foraging decisions (e.g., about where to forage for food) can be cast in terms of Bayesian inference, as the computation of Bayesian posterior beliefs from prior beliefs about the base rates of occurrence of relevant factors in the niche and sensory evidence about what the current situation is. In the case of the colony, this Bayesian estimation is implemented through chemical stigmergy and tactile interactions among nestmates within a situated niche. Colonies that fail to forage sufficiently (or over-forage and risk desiccation/predation) will quickly fail, in a process analogous to Bayesian model comparison and reduction at the population level.

The Bayesian framework has been applied extensively to foraging and non-foraging behavioral paradigms in multicellular organisms, and also in the brain (Friston et al., 2014; Ramstead et al., 2019b; Russell-Buckland et al., 2019). The multiscale Bayesian framework maps well onto the eusocial insect colony, since eusocial colonies are the unit of function that is shaped by natural selection (reflecting changes to the multiscale or multilevel inferential scheme) (Wheeler, 1911). Functional similarities between ant colonies and Bayesian inference schemes also include ability to engage in long-term self-organization, self-assembling, and planning, by implementing highly nested cybernetic architectures (dense heterarchies; Wilson and Hölldobler, 1988; Fernandes et al., 2014; Friston et al., 2018; Friedman and Søvik, 2019; Friedman et al., 2020a). By integrating the “Bayesian ant colony” perspective (Hunt et al., 2020a) with stigmergic regulation and the scale-free formalism of active inference (Fields and Glazebrook, 2020) we provide an integrated framework for behavioral, ecological, and evolutionary modeling in ants (Ramstead et al., 2019a). Such an integrated approach could facilitate the transfer of modeling insights from ant colonies to human-designed systems. Akin to other recent modeling work in active inference in areas such as machine learning and human interoception (Sajid et al., 2019; Smith et al., 2020), a goal of the work here is to demonstrate areas of resonance between an active inference perspective on the ant colony and other previous approaches [e.g., the long history of ant colony simulations and optimization techniques (Blum, 2005; Dorigo and Stützle, 2019), and especially recent Bayesian work such as (Hunt et al., 2020a)]. With this mapping in hand, ant colony modeling could benefit from parallel developments of the active inference framework into areas such as nested multiscale modeling, learning and memory, ensemble learning, and robotics.

### An Active Inference Model of an Ant Colony

In this paper, we develop and implement a Bayesian simulation model derived from the active inference framework that captures some of the underlying dynamics and emergent phenomena of ant colony foraging behavior. In our model, we focus on the *stigmergic* outcomes of foragers using a single trail pheromone molecule. Assuming that each agent (nestmate) only has limited access to incoming information, how can a group of active inference agents solve complex group foraging problems?

The active inference framework naturally lends itself to modeling behavior of different systems across scales. Generally, what changes across models available in the literature are agent action affordances (e.g., visual scanning vs. physical movement), the semantics of the prior beliefs (i.e., what it is about, the type of generative processes/models used), and the hyper parameterization (i.e., the prior distribution over beliefs). From the point of view of the dynamics (i.e., the message passing and update equations) all active inference models are the same. Here we present the first Active Inference type model of stigmergy, and the first Active Inference model focusing on insect behavior. Our simulation adapts the inferential architecture developed by Constant and colleagues (Constant et al., 2020) to the setting of ant colony foraging. The Constant et al. model studied the “visual foraging” of a single agent while it scanned patterned artifacts. The novelties and distinguishing features of our work are several-fold. First, we adapted into a different sensory modality (chemosensation rather than visual). Second, we considered a multi-agent simulation rather than modeling the foraging activity of single agents. Lastly, we introduced the ability of homeward-bound ants to deposit trail pheromone (an affordance inaccessible to visual foragers), and thus brought the model into the stigmergic context that is natural for the ant colony system.

Relative to other agent based simulations of ants, the advantage of the presented simulation is its interoperability with other models of similar type, and the fact that many environmental and biophysical manipulations can be performed using the same base model (see code repository for this paper and other contemporary work using similar active inference model structures). Thus unlike custom ant-specific models coded in various languages, having an abstraction for ant colony function based on active inference agents can be transposed into different contexts. The biological reality of ant colony foragers is that they have evolved various features such as learning, neuromodulation of sensitivity to pheromones, the ability to leave multi-component pheromone trails, the ability to maintain a given colony size, regulate colony foraging, etc. Even though the proof of principle on offer does not model these features explicitly, all these features could be explored under a future active inference model. We come back to this point in the discussion section.

In the model presented here, foraging ants move around in a defined area that includes a nest location and a food resource location. The forager actively infers a single sensory (gustatory) outcome: an idealized attractant trail pheromone (Saad et al., 2018; Friedman et al., 2020a). This pheromone trail cue, modeled as a preferred state in active inference framework, is discretized into 10 levels (reflecting differential concentration/deposition of the idealized pheromone). The sensory outcomes that represent pheromone trails are biologically grounded in the oily glandular compounds of varying volatility that are deposited on surfaces by ants while inside and outside the nest (Leonhardt et al., 2016; Stökl and Steiger, 2017). Over 168 trail pheromones have been identified in the literature (Cerdá et al., 2014) and they likely act in redundant or combinatorial ways.

The T-maze choice test and related Y-maze paradigm (Oberhauser et al., 2019) are classical laboratory paradigms for studying the decision-guided behavior of foraging in various systems, including mammals (Iodice et al., 2017), solitary insects (Jiang et al., 2016), and ants (Czaczkes et al., 2013, 2015a; Czaczkes and Heinze, 2015; Saar et al., 2017). The T-maze test variant known as the T-maze alternation test involves switching the location of a food source or reward cue between arms of the T-maze, with some specific pattern through time. In mammals, alternating T-maze tests have been used to investigate topics such as learning, memory, decision-making, and preference (Deacon and Rawlins, 2006; Shoji et al., 2012). As for T-mazes in ants, Czaczkes wrote that “T- and Y-mazes are powerful tools for studying the behavioral ecology and cognition of animals, especially ants” (Czaczkes, 2018), motivating the use of the T-maze paradigm here. The alternating T-maze paradigm allows exploration of the interplay between the individuated cognition of nestmates and the shared, stigmergic, distributed cognition of the colony (*via* pheromone deposition here in this model).

To assess colony foraging behavior in a well-studied laboratory paradigm, we simulate our *in silico* ant colonies as central-place foragers in a T-maze behavioral paradigm. We conclude with a discussion of how one could build upon the proposed simulation to inquire about the evolution of multiscale foraging processes in ant colonies and other Bayesian agents, to understand how these complex systems solve various challenging problems with limited and local information.

## Methods

### The Foraging Task

In our model, foragers leave the nest and engage in random movement, biased by the concentration of a locally-detectable attractant trail pheromone. On the way out of the nest, the unladen forager does not lay down any trail pheromone. Once a forager discovers the food resource (detects the scent of food), it begins depositing trail pheromone as it navigates back to the nest entrance. This usage of attractive trail pheromone by incoming successful foragers is inspired by the ecological context of foraging in the *Formica* red wood ants: “Evidence is presented that the cause of directional recruitment in *F. rufa* group ants is a scent trail laid from the bait toward the nest, while ‘centripetal' recruitment, due to orienting signals provided by scouts returning to the bait from the nest, is of negligible importance” (Rosengren and Fortelius, 1987). This strategy of depositing attractant trail pheromone only on the way home after a successful foraging trip, is in contrast with aggressive explorers such as the Argentine ant, which can lay down attractant trail pheromone on the outbound and thus recruit rapidly (Flanagan et al., 2013). In addition to the single attractant trail pheromone used here, this model of colony foraging includes a food scent [e.g., the scent of a seed (Gordon, 1983; Greene et al., 2013)], and a “home” scent [reflecting the unique pheromone profile of the nest and nearby soil (Johnson et al., 2011; Huber and Knaden, 2018)].

The basic challenge faced by the ants in our simulation is to locate the food resource patch, which alternates every 500 time steps in the simulation (representing a transiently-available, patchy resource) over 2,000 steps. The colony must rediscover the food location (on the other side of the T-maze) when its location changes. We add a decay parameter to the attractant trail pheromone, such that at each time step, the density of the pheromone at the visited locations decays with a small probability (0.01% in the simulations presented here). Biologically, this can be viewed as the gradual decay or evaporation of lipid pheromones (which range from volatile to wax-like). Informationally, this decay rate can be thoughts of an environmental perturbation or drift that counteracts the reinforcing effect of stigmergic convergence.

In our simulation, after discovering the food, inbound foragers begin a random walk that is biased toward higher concentrations of trail pheromone and also biased toward the direction of the nest entrance. The biological plausibility of this inbound turn is well-supported by a variety of mechanisms used in ants to navigate home from foraging trips, including the path integration of steps (Heinze et al., 2018), location of sun and moon in the sky (Wehner and Müller, 2006; Freas et al., 2017, 2019a), magnetoreception (Freas et al., 2019b), recognition of remembered visual scenes (Baddeley et al., 2012; Zeil et al., 2014), interactions with nestmates on the trail, non-pheromonal olfactory cues (Steck, 2012), the geometry of the trail network (Collett and Waxman, 2005; Czaczkes et al., 2015b), and so on. Because successful foragers deposit trail pheromone on their inbound trip, they act to reinforce extant pheromone trails.

In this paper we show results from simulations with several different numbers of foragers (10, 30, 50, 70), however with the provided code one can adjust this to any value. We performed simulations of 2,000 time steps (this value also can be set to any number), meaning that each ant carried out 2,000 steps of action and inference. We considered two summary measures as colony-level phenotypes.

First a colony foraging performance metric which is simply the number of round trips between nest and food location over the 2,000 time steps. The more round trips that are completed per unit time, the better task performance is, in the sense that successful foraging was accomplished more often. As the location of the food resources shifts several times during the simulation (at three times: 500th time step, 1,000th time step, and 1,500th time step), successful colony foraging here represents an ability to both discover and exploit resources that are dynamically changing through time.

Second a swarm coherence metric was calculated as a distance measure among ants at each time step as:

$$\begin{array}{l} \text{Distance Coeff}=\frac{1}{N_{ants}}\overset{N_{ants}}{\underset{i=1}{\sum }}dis\left(ant_{i},\;ant_{j}\right)\;for\;j\;=\;1,\ldots ,\;N_{ants} \end{array}$$

The distance coefficient quantifies the average distance between all ants at any given time point, where *dis* is the Euclidean distance between two ants, i.e., $\sqrt{,}$ with *x*_*n*_ and *y*_*n*_ denoting the *x* and *y* position of ant *n*. The goal of this simple swarm distance metric was to capture some possible signatures of collective phase transitions (for example assuming even mixing on the north-south axis of the map, a reduced distance metric would imply that all ants were on the same arm of the T-maze, while increased values would suggest distribution on both arms).

### An Active Inference Model for Ant Colony Foraging

Active inference is a Bayesian theory of behavior that accounts for perception, learning, decision-making, and the selection of contextually appropriate actions, which are all cast as a form of approximate Bayesian (or variational) inference. In this framework, perception is modeled as the formation of posterior state estimates, which are arrived at by combining priors and likelihoods, that is, the prior beliefs had by an agent about the base rate of occurrence of states and the likelihood of making this or that observation given this or that states; learning is cast as the process of updating of priors, likelihoods, and other model parameters; and decision-making and action are cast as processes that compare the evidence for various models about future states and observations under possible courses of action (see Appendix).

**Figure 1:**
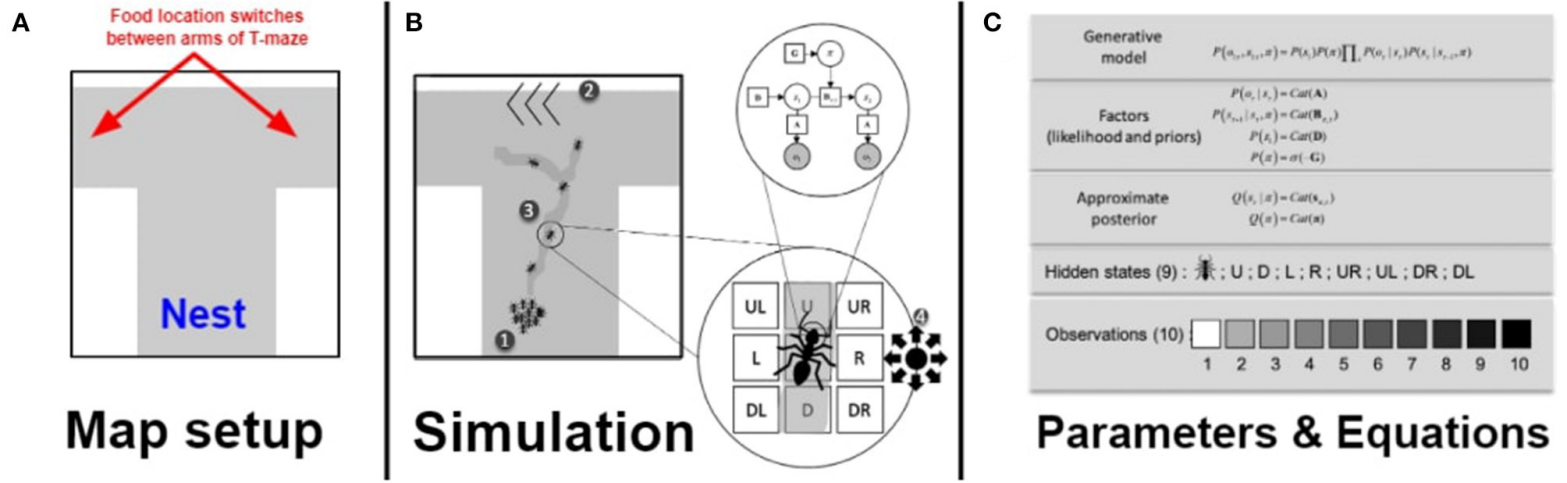
Overview of the foraging paradigm and simulation presented in this paper. **(A)** In this paper we used a T-maze foraging paradigm. Foraging nestmates issue from the Nest location in blue at the bottom of the T-maze and the food switches location between the arms of the T-maze during the simulation. The gray shaded area is where ants can forage within. **(B)** During the simulation, described in the order of the labeling of the figure: (1) All agents (simulated ants) start at the same nest location. (2) Their task is to reach a food resource on the map, which is on one arm of a T-maze. (3) While unladen (e.g., heading out on a foraging trip), the ant does not deposit pheromone, and moves toward locally increased density of pheromone. After encountering the food, the ant leaves a pheromone trail on its way back toward the nest, again moving toward locally increased density of pheromone. (4) Crucially, ants only have sensory access to their immediate surroundings and do not know where the food resource is located on the map. All they can sense is what surrounds them immediately and its current location, as well as outcomes associated with each of these states. **(C)** Expanded table of Parameters and Equations. For each ant, at each time step, there are 10 possible observations, which correspond to 10 levels of pheromone density (which range from 1 to 10). Each ant can select policies that transition between surrounding locations (which are modeled as hidden states that the agent has to infer based on sensory cues). This means that the generative model of each ant is made of a likelihood and a prior that are limited to the representations of these nine locations. The policies available to the ant correspond to transitions from the current location to one of nine possible locations (i.e., same location, up, right, down, left, top-left, top-right, bottom-left, and bottom-right), and evolves over one time step. (5) The environment in which the ant navigates is a 40 × 40 matrix, with each location corresponding to an index from 1 to 1,600 that allows us to respecify the likelihood matrix of the ant after each step (see Appendix). Each location in the environment generates a given outcome, which can change based on whether the ant passed over that location and left a pheromone trail. There is no modulation of the likelihood precision in our simulation, which means that each ant always has an unbiased sensory access to its surroundings.

$17 The simulation uses a Markov decision process (MDP) formulation of active inference; for details, see the code and (Friston et al., 2016). Here, it is important to understand that the MDP corresponds to the decision process of a single simulated ant forager, not the colony as an entity. Indeed, the MDP corresponds to the sort of cognitive inferential process that a nestmate ant would have to undergo in order to select a course of action or policy. The Bayesian generative model is a joint probability density over hidden states and observations that decomposes into several factors (Figure 1). These are (i) the likelihood of observations, denoted **A**, which specifies the probability of observations given hiddens states; (ii) the transition probability matrix, **B**, which harnesses the probability of state transitions, i.e., the probability of transitioning from one state to the next, given some action. **C** is a preference parameter that foragers have for denser pheromone traces, i.e., the agent prefers to move in the direction of the locally-densest pheromone trail. The prior over initial states after each cycle of inference is denoted **D**. Finally, prior preferences for data **C** enter into the computation of the expected free-energy, **G**, which is a vector of values that corresponds to the free energy expected under a given course of action or policy through time (normalized by a softmax function).

Variational inference is a method for approximate Bayesian inference and depends on two distributions: the variational distribution and the generative model. The variational distribution is a distribution over all unknown variables (states and policy) in the model and represents the agent's beliefs about the current state of the world. The generative model describes an agent's “model” of the world, and specifies a mapping from hidden states to observations. Variational inference then looks to invert this mapping and recover the mapping from observations to hidden states (see Appendix and code for more details on the equations used in the variational approach used in this paper). Here we use the active inference model in an instrumental capacity [e.g., as a statistical apparatus that is useful for addressing questions about the world (Bruineberg et al., 2020)] rather than in a realist fashion to say what ants are “really doing” given our observations (Gordon, 1992).

The generative model of each ant consists of a single hierarchical level (Figure 1). The hidden states correspond to eight possible locations that surround the ant at any given time, plus the ant's current location. The possible observations (*o*) of the ant are the intensity of pheromone, ranging from 1 to 10. This pheromone intensity reflects the hidden underlying actual pheromone density at each location (*s*). The likelihood parameter (“**A**”) parameter maps all possible sensory outcomes (10) to all accessible locations at each given time. The state transition probabilities are coded as the transition parameters (**B**_**π, t**_), which encodes all possible transitions among all possible states, conditioned on policies (π, sequences of actions). Effectively, this means that there are nine transitions matrices, for nine possible policies that allow the ant to go in all directions. The likelihood and the transitions are fixed in all simulations. We use this generative model and an initial state distribution **D** to generate sequences of potential future outcomes and hidden states and potential 1-time step policies (sequences of action). Policies are scored in terms of their expected free energy (denoted **G**). The expected free energy of each available policy is evaluated (here the accessible cells), and the policy with the least expected free energy is selected by the agent. Preferences are encoded in a vector **C** that specifies a desired probability distribution over outcomes. To generate the different phenotypes of Supplementary Files 6, 7, we change the values in the preference vector. In this context, flat priors simply mean that the agent has no preferences for any particular observation, i.e., it believes all observations are as likely and thus as preferable. In contrast, we use strict priors to refer to the case where there is a monotonic increase in probability as a function of pheromone level, i.e., higher levels of pheromone have higher probability, and are thus more preferable. As per the instrumentalist reading above (Bruineberg et al., 2020), these model parameters are variables within a modeling framework, not hypothesized or proposed to be specific neural or cellular features of real ants.

Note that the model on offer differs from some active inference MDP models in that the agent—here, the ant nestmate—does not have an internal representation of the complete structure of the environment (i.e., a map with associated sensory outcomes). Here, the ant can only represent the pheromone gradient which surrounds it locally and instantaneously. Specifically in our grid automata model, the forager can detect one location around its current location in the map, in each direction (up, down, left, right, top-right, top-left, bottom-right, and bottom-left). These local pheromone deposits make up the hidden states. For real ant foragers, the perception horizon for chemosensation is limited by the reach of their antennae (ants cannot detect smells “at a distance,” only smell what is immediately local). Additionally the ant can only take steps in their immediate direction (cannot teleport).

To limit the perception and inference horizon of the ant in a biologically realistic way, we added to the standard active inference methods a recently-developed “reallocation matrix” (see the Appendix). To ensure that the ant only was in possession of local access to its sensory environment (i.e., the 10 levels of pheromone density), we utilized a likelihood remapping strategy that respecifies the entry of the likelihood matrix after each time step (see Supplementary Figure 1). Likelihood remapping allows the agent to perform state inference and navigation by bypassing the full representation of the generative process (i.e., environment). This is in contrast to standard active inference approaches which typically require the agent to be given a correct global understanding of the scene. To achieve this locality, the “A” matrix becomes state-dependent so that it only provides information about outcomes in the proximity of the state the agent is in. The reallocation matrix itself corresponds to the size of the environment (here 40 × 40, or 1,600 locations), and maps each possible observation to its location in the environment. In that sense, the reallocation matrix can be viewed as a likelihood matrix for the environment – [P(pheromone | location)], (see Appendix), or generative process from which we manually sample to specify the likelihood of the ant at each time step. Crucially, once the ant selects an action, it then moves in the environment, and based on where the ant is at that step, we remap the likelihood of the ant for the next cycle of policy selection. Pheromone trails are then “deposited” in the reallocation matrix to change the distribution of outcomes (i.e., pheromones) in the environment, thereby allowing for stigmergic interaction between all ants *via* the reallocation matrix.

To summarize the computational activity of each forager at each timestep, pseudocode is provided here (see Code for full details). The active inference model as implemented here does not presuppose or imply any specific neurocognitive architecture for any specific ant species. At each timestep of the simulation, the agent:

1. Senses environment (local pheromone density)
2. Optimizes beliefs with regard to Variational Free Energy (VFE, Appendix Equation 3)
3. Optimizes beliefs about action with regard to Expected Free Energy (EFE, Appendix Equation 4)
4. Samples action from beliefs about action (performs action selection)
5. Agent performs action (movement) and updates the environment (deposits pheromone, if laden with seed)

Our unimodal (gustatory) model here does not take advantage of forager visual capacity or interactions, and only is based upon the stigmergic principle of pheromone deposition. Future models could include ant bodily state, development, memory, and intermodal integration. The reallocation matrix allows us to remap the likelihood matrix of the generative model of the ant based on its movement in the environment (it is a movement policy matrix). The purpose of the reallocation matrix here is to allow such a remapping of the likelihood of the ant, which is just a 3x3 portion of the reallocation matrix relative to where the ant is at a given time step. In other words, this reallocation matrix is what transforms the global computational representation of the foraging arena, into a locally-accessible space around each ant, guaranteeing that the relevant information used by each ant is really only local.

Stochasticity in decision-making at the nestmate and colony level may facilitate adaptive behavior in ants (Deneubourg et al., 1983). In our model, stochasticity enters into the picture at the model step where policies (movement directions) are sampled from the distribution over all policies based upon their relative expected free energy. Thus, in the absence of any spatial pheromone gradient (or in the case of a flat preference for pheromone density), the ant forager diffuses according to Brownian motion on a grid (e.g., equally likely to go any direction). If an argmax were used rather than a sampling-based approach, this model at each time would be fully deterministic (but even in this case it would not entail the colony outcomes being easily predictable due to complex stigmergic dynamics). Additional kinds of stochasticity could be implemented at various steps in the model, for example by having imprecise sensory input. Interactions among ants and other parameters guiding search behavior could be added as well (interactions, momentum, celestial radiation, other odor cues, etc.), this model seeks to frame the gustatory components of ant colony stigmergy in terms of actively inferring agents.

## Results

**Figure 2:**
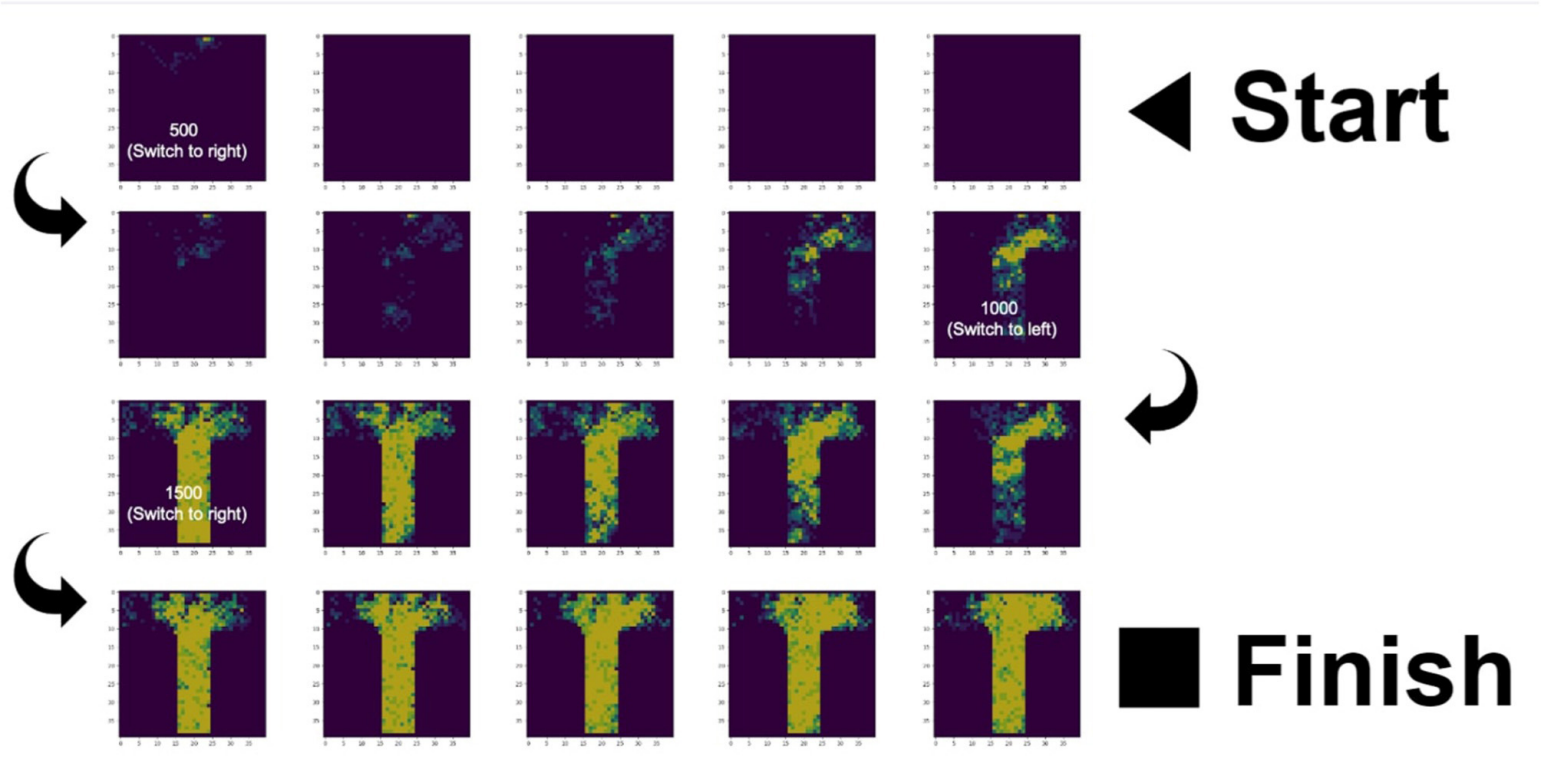
Pheromone trails of the colony with 70 nestmates over 2,000 timesteps and three food location switches. The sequence of images starts on the top right and reads from right to left (1st row), from left to right (2nd row), from right to left (3rd row), and from left to right (4th row). We chose a representative simulation and did not tune the parameters to discover optimal decision making. See Supplementary Files 6, 7 for example simulation outputs and to see the switching behavior (pheromone density locking onto one arm, then gradually locking onto the other arm) that is observed in the case of strict priors but not flat priors.

The simulation ran successfully: ant colonies consisting of foragers with no internal map of the T-maze were able to forage successfully given a set of simple behavior rules (nestmates pursue locally increased pheromone density using an Active Inference model, and lay down pheromone when returning home). Figure 2 shows screenshots for the 70 ants colony at a few timepoints, displaying the colony at stages of searching, exploiting, and switching. For animated .gif's of simulations of different colony sizes, see Supplementary Files 1–7 and the code availability statement.

### Colony Foraging Performance

**Figure 3:**
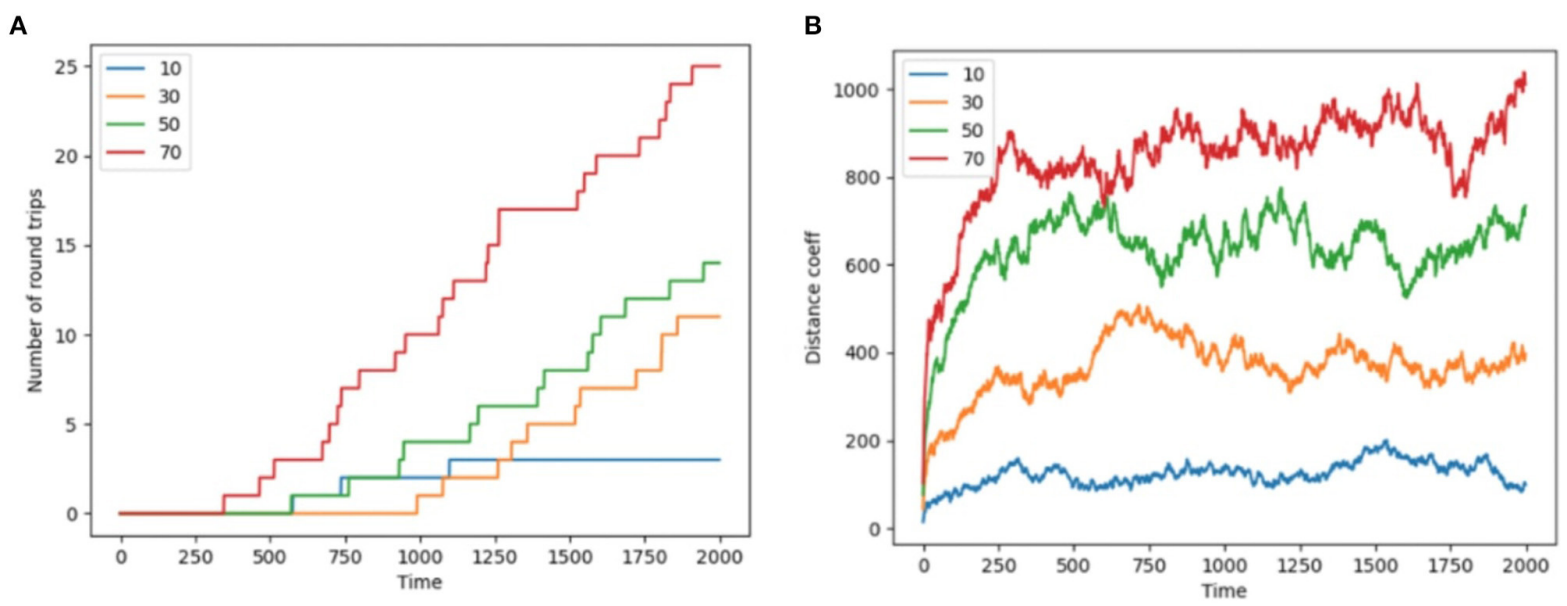
Round trips and distance coefficient. **(A)** The number of completed round trips (Y axis) through time (X axis) for different numbers of nestmates in the simulation (traces of different color as per legend) for a representative trace run of each colony size. **(B)** Swarm distance coefficient (Y axis, see section The Foraging Task for equation) plotted through time (X axis) for different numbers of nestmates in the simulation (traces of different color as per legend) for the same representative runs.

Colony-level phenotypes are summary measurements of emergent properties in that these phenotypes do not apply to separate nestmates (though they are enacted by nestmates; Feinerman and Korman, 2017; Gordon, 2019; Friedman et al., 2020a). For example each nestmate has bodily phenotypes (such as morphology and tissue-specific gene expression patterns), but colony phenotypes are those that only exist at the colony level (e.g., total foraging performance, average inter-nestmate distance), Figure 3 shows two measures of colony foraging phenotype: the total number of foraging round trips completed though time, as well as a swarm coherence metric (see Methods section The Foraging Task for equations defining the swarm coherence metric).

First, as a measure of colony foraging performance, we recorded the number of successful foraging trips, i.e., round trips from nest to food location patch to nest performed by each ant in each simulation through time. We found that colony size influences the number of round trips taken per-nestmate over the simulation's duration (Figure 3). The influence of colony size on round trips appears to be non-linear in that the colonies of different size are not simply scalings of one another. However we do not draw any generalizations from this specific trend, since we did not explore variation in critical model parameters such as T-maze size, pheromone deposition rates.

Additionally our model did not include fundamental types of information relevant for real foragers, such as the pattern of interactions among nestmates, smells other than attractant pheromone, or the location of celestial lights. Across all colony sizes explored in our simulations, foragers were consistently able to locate the target resource at the distal end of the T-maze and recruit nestmates using stigmergic pheromone deposition. Research on colony foraging metrics has been carried out in many ant species globally, and in the laboratory many behavioral paradigms have been used. Here we only made basic summary measures of colony foraging performance (Figure 3), with an eye toward the kind of measurements that could be quantitatively explored in future simulations and empirically inferred from ants in the lab and field.

### Swarm Coherence

Another colony-level phenotype or summary measurement is the degree of coherence among nestmates. This can be thought of as the “stigmergic tightness” of the system, in that tightly-regulated stigmergic systems might show lower average inter-agent distances, while looser systems would have higher inter-agent distances. For representative simulations, Figure 3B shows the change in the distance coefficient between ants, at each time step, over 2,000 time steps, for each colony size (10, 30, 50, 70). For each colony size, the colony quickly converges onto a characteristic range for the distance metric (Figure 3B e.g., ~100 for colony size of 10 and ~900 for a colony of 70). By looking at swarm coherence metrics through time, it could be possible to identify phase transitions and regimes of meta-stability for swarms.

Here we also found that colony size influenced swarm cohesion metrics, and will explore this space in a more statistically- and ecologically-informed fashion in future work. Specifically one could ask how distance- and trajectory similarity-based measures of stigmergy might capture characteristic or functional attributes of colony behavior, or the manner in which various environmental challenges (e.g., food location switches frequencies and maze shapes) in such a characteristic summary distance metric. Swarm coherence metrics might also be defined not in terms of inter-agent distance, but rather the correlation, mutual information, or degree of stereotypy among trajectories.

## Discussion

Here we have presented an active inference framework to consider some aspects of the multiscale and emergent dynamics of an ant colony. Active inference may be informative in the study of ant colonies at three distinct temporal scales: nestmate behavior (emergent among tissues of a nestmate and interactions with the biotic/abiotic environment), colony development (emergent among nestmates and their interactions with the niche), and the evolution of populations (emergent among colonies within an ecology). In this work, we have focused on the connection between the first two levels of analysis, specifically focusing on the case of stigmergic colony foraging, using a modification of previous active inference computational implementations. We utilized a partially Markov decision making process model based upon previous work, with several ant-specific modifications (Methods, Figure 1).

We found that colonies of active inference foragers were able to discover and stigmergically exploit food resources in a simple T-maze (Figure 2). Foragers engaged in a local search for directions with more deposited pheromone, and added pheromone to tiles as they returned to the central nest. In this work we explored a few metrics of colony performance such as overall colony number of round trips, and swarm coherence metrics (Figure 3). We did not perform extensive parameter sweeps (e.g., to optimize performance or draw generalizable conclusions across dimensions of variability). Our model lacks several important features that influence colony foraging activity in realistic settings (such as tactile interactions and physiological heterogeneity among nestmates). There are three levels of dynamics of ant colonies that could be simulated in the future.

### Nestmate Scale: Behavior and Development

The first scale of analysis to consider in the ant colony case is that of perception, learning, and decision-making under active inference for ant nestmates (i.e., a nestmate behavioral model). This is the scale that we explored here in this series of simulations. We limited our simulation work to perception and a rudimentary form of hyperlocal decision-making (nestmates only do inference on the next timestep and immediate spatial surroundings). In so doing, we did not take advantage of the full scope of active inference. Active inference can model phenomena such as curiosity, novelty, affect, and valence biases in terms of how they arise from estimates of world states and variabilities (Parr and Friston, 2017). In the context of ant navigation and foraging behavior, learning such kinds of statistical regularities might have to do with learning of affordances (available actions) and statistical regularities in the terrain [e.g., a bifurcating node on a tree trunk might afford the nestmate the possibility of moving to one side or the other (Chandrasekhar et al., 2018)]. In a completely flat environment such as the one we simulated, there is no uncertainty in transitions from one location to the next (all local steps can be taken). However, learning volatility may be important for adaptive navigation and might be an interesting manipulation to augment our existing simulation setup in future experiments. Models at this level of simulation could also include neurologically-plausible implementation in terms of the mid-sized neural architectures in some insect brain regions (Cope et al., 2017; Müller et al., 2018).

Learning under active inference is another feature that we did not explore here and that may be interesting to simulate in ant colonies, especially the learning of precision parameters that have been previously associated with dopaminergic neuromodulation (Friston et al., 2014). Across eusocial insect species, brain dopamine titers appear to be higher in foragers than inside workers (Friedman and Gordon, 2016; Kamhi et al., 2017), and pharmacological increases in dopamine signaling stimulate foraging behavior (Barron et al., 2010; Søvik et al., 2014; Friedman et al., 2018). Other neurotransmitters such as tyramine and serotonin are also important for regulating nestmate foraging activity, perhaps by modulating sensitivity to sensory cues (Muscedere et al., 2012; Scheiner et al., 2017). Thus it could be the case that with insects as with vertebrates, dopamine and related neurotransmitters play key roles in modulating the precision of state estimation, thus regulating a wide variety of goal-seeking and decision-making behaviors (Barron et al., 2010; van Lieshout et al., 2020). Full neurocomputational models exist for navigation and multisensory integration in foragers (Cope et al., 2017; Goldschmidt et al., 2017) and would be interesting to integrate with agent based active inference models of the type presented here.

Under the classical behavioral framing of ant foraging behavior, the “reward” is defined as the target food resource (e.g., seed, sugar water), and the pheromone trail is considered as a semiotic cue that guides the forager toward the target. Under the active inference framework, the reward (preferred observation) plays a more semiotic role (in engaging a switch in the foragers behavior from outward to inward bound) while the pheromone trail density plays the role of a reward (in that it is what is being pursed locally by the forager). In terms of observations about the pheromone trail, and the target resource is a cue that engages a switch in course of action (i.e., selecting a course that is outward bound vs. inward bound). Acting as a function of reward, here, just means selecting the action that provides the most evidence that the agent is generating preferred observations [for details see Friston et al. (2009) and Costa et al. (2020)]. This means that what motivates the active inference forager to move toward the target that is adaptive for the colony (e.g., some tasty seed) is a preference for pheromone trail density, i.e., it accomplishes foraging by pursuing high levels of pheromone. Heuristically, in each cycle of policy selection, the simulated ant selects an action that brings it where it believes pheromones concentration will be densest. The idea is that because other successful nestmates have left pheromone traces as they returned from the food resource, for an outbound forager, performing policy selection based on pheromone preferences will increase the success of finding a trail that connects the nest entrance to the resource. When the food location changes (as it does every 500 timesteps in the simulation we present here), pheromone trails connecting the nest entrance to the now-vacant food source will decay, but the trunk of this trail will be reinforced once the food is discovered on the other arm. Thus the nestmate pursuit of high local pheromone density, coupled to an adaptive policy of “add pheromone only when successful and returning home,” is sufficient to enable effective colony search. In evolutionary intergenerational simulations, this forager preference distribution could be tuned, and colonies with successful forager preferences would have increased survival and reproduction. Ant nestmates may carry out foraging behavior using similar neural elements involved in the regulation of foraging in solitary insects (Barron et al., 2010; Landayan et al., 2018), as well as novel regulatory components due to the distinctly different case of the eusocial nestmate forager life history in terms of inputs, regulation, and outcomes (Friedman et al., 2020a).

### Colony Scale: Stigmergy and Extended Cognition

The second level of dynamics here, not addressed in our simulation, is the learning of nestmates and development of colonies. The colony as an organism itself has developmental phases that go beyond the timescale of behavior (Wheeler, 1911). Such colony-level development includes changes in size and worker task or morphological specialization. Additionally colony-level development can encompass niche construction phenomena such as nest architecture. This timescale is not simulated here, but is a topic for future work.

The representation of the environment leveraged to solve the T-maze problem—i.e., the “map”—is not one that is entertained by nestmate brains, but rather one that is carved out of the environment and leveraged *implicitly* by each ant as they each forage alone, together as a colony, leaving cues that nestmates can use adaptively. In active inference terms, the ants are endowed only with an *extended generative model* (Constant et al., 2019a). An extended generative model is one where some of the model parameters are encoded not by the agent engaging in inference itself, but by the physical environment in which it lives. This form of self-organized behavior is common in social animals, such as human beings. For example, traffic signals can be viewed as mechanisms that allows agents to offload the processing of information related to their peers' driving behavior onto the structure of the environment; agents no longer have to anticipate the behavior of peers (or at least, much less so), and only have to follow the cues provided by traffic signals, which indicate what is appropriate behavior in a given situation (Constant et al., 2019b; Veissière et al., 2019). In the present study, these active inferants can only represent information in their immediate surrounding, for example, pheromone patches with more or less density. They rely on information encoded in the environment itself (i.e., pheromone trails) to guide their behavior. Importantly, the agents are not learning a world-centric map-like representation of where the food is. In this modeling framework, foragers are acting probabilistically, given their preferences and beliefs about their local environment or peripersonal space.

The model presented here only uses a single attractant pheromone and this could be expanded through different sensory inputs to consider mixtures of chemicals. Ecologically, in cases where it would be advantageous for a nestmate to visit a location where previous nestmates have been, the use of an attractive pheromone is found (Butler et al., 1969; Lanan, 2014; Feinerman and Korman, 2017), while in situations where it would be disadvantageous for nestmates to visit locations where other nestmates have recently been, chemical cues are commonly repulsive (such as alarm pheromones; Wilms and Eltz, 2008; Hunt et al., 2020a). The model could include a repulsive pheromone as well as an attractant, and foragers could have complex or learned preferences related to how they differentially weigh the pursuit of attractant gradient vs. the evacuation from repulsive gradients. Additionally, variation among colonies in collective behavior may arise from patterns of physiological variation among nestmates of the same or different developmental stage (Gordon, 2019; Lemanski et al., 2019; Friedman et al., 2020a). By performing statistical analysis on the variation among nestmates in interactions and foraging activity, the model could explore the role of nestmate variation in response threshold (Yamanaka et al., 2019) in colony performance and variation among colonies in behavior (Friedman et al., 2020b).

In ecological settings, ant colony behavior is regulated through multi-component pheromone gradients as well as dynamic interactions among nestmates. Collective behavior is an ecologically important feature of ant colonies and a target of selection. Only foragers were included in this model, future derivations could include worker developmental trajectories and colony-level task allocation processes (Friedman et al., 2020a; Hayakawa et al., 2020). Nestmate-level behavioral heuristics are important for colony efficiency (Gordon et al., 2019; Kamhi et al., 2019; Arganda et al., 2020), as are truly colony-level processes (e.g., dynamic interaction patterns and nest architectures (Gordon, 2010; Pinter-Wollman et al., 2018; Lemanski et al., 2019) are in tight feedback with nestmate-level development and task allocation. Our model could be extended to study the role of these processes in the evolution of colony behavior, since the proposed simulation involved only agent-environment stigmergic interaction. These interactions among nestmates could be investigated using this setup by adding in an effect of spatial encounters among ants (i.e, collective behavior based on a shared physical environment or substrate; Razin et al., 2013; Davidson et al., 2016).

In ants, the extent of neurophysiological variation among nestmates (in e.g., sensitivity to interactions or response threshold) may contribute to variation among colonies in behavioral outcomes (Lubertazzi et al., 2013; Lemanski et al., 2019; Friedman et al., 2020b). In this simulation, all ant nestmates had identical sensitivity to the pheromonal cue. Future simulations could implement nestmate-specific sensitivity parameters, which for example could change in response to development or sensory experience. A benefit of the Markov decision is that it does not presuppose any specific neural or cognitive mechanism within the nestmate brain. The key specifications of the model are in terms of what can be sensed (a single attractant pheromone in this case) and what actions are possible [here only walking, as per other models (Wilensky, 1997)]. Using recently developed immersive technologies for ants [such as the “Antarium” (Kócsi et al., 2020)], it could be possible to combine observation in natural settings, simulations, and laboratory experiments to understand neuroethological aspects of ant foraging.

In this model we considered a single ecological regime (e.g., the food location was dynamic, but we only explored one rate of food movement). We did not, for instance, manipulate several key parameters of the model that might have influenced the number of round trips between the food location patch and the nest. Also of note is that eusocial insect foragers are able to communicate information to each other regarding foraging resources through interactions and chemical stigmergy. This means that foragers are able to distinguish and integrate various internal and external factors related to bodily physiology (Silberman et al., 2016) and memory (Stroeymeyt et al., 2011; Oberhauser et al., 2019). In other words, while in the model presented here each nestmate was functionally similar and memoryless, future models could have functional and developmental variation among nestmates, for example to address questions related to how foragers manage the tradeoff between shared/private information and distant/recent experiences. Such scenarios can be specified based on the proposed simulation, and manipulations can be empirically validated. Along the lines of complex systems modeling approaches such as cadCAD (2020), digital twin simulations of ant behavioral experiments would allow determination of statistical power, pre-registration of experiments, and detection of relevant experimental parameter ranges for empirical implementation.

### Population Scale: Evolution and Ecology

The third time scale of dynamics of the ant colonies, also not addressed directly by this model, is evolution: the intergenerational process which acts at the level of a population of colonies. Selection shapes nestmate-level behavioral parameters by shaping neuromodulatory physiology and other features of sensorimotor processing (Gordon, 2014, 2016). In this casting, natural selection is akin to the process of Bayesian model comparison or reduction, an efficient technique for model comparison (Alhorn et al., 2019; Smith et al., 2019). Selection favors colonies where nestmates enact models that, under ecological challenges faced by similar models (colonies) in the population, are more adaptive (Wilson and Hölldobler, 1988; Gordon, 2014; Boomsma and Gawne, 2018; Friedman et al., 2020a). This adaptive optima for foraging is related to many other tasks and traits, and optimizing it does not mean maximal foraging rate. The most adaptive tradeoff on a complex multidimensional fitness landscape includes the costs of e.g., desiccation, predation, and false alarms. It is especially interesting if selective processes are able to shape colony- and nestmate-level learning rates (e.g., learning volatility), because it allows one to compare models that are adapting to different environmental niches over multiple timescales.

## Concluding Remark

The active inference framework provides a template for future work on foraging behavior, to incorporate temporal variability, learning and memory, multiple types of pheromone, abiotic factors, interaction patterns among nestmates at different developmental stages, and population-level processes. Based on the proposed model, future research could explore several key axes of ecological variability (e.g., patchiness of resources) and recover some of the classic findings of laboratory and field behavioral observations in insects. The ant colony has long been an inspiration for agent based models and robotics, as well as computational algorithms (Rossi et al., 2018; Dorigo and Stützle, 2019). With the introduction of active inference into this space, it could be possible to bridge from these engineering fields, to more abstract areas such as information processing in networks, cybernetics, and collective graphical models (Sheldon and Dietterich, 2011; Baluška and Levin, 2016; Fekete, 2019). The Hymenopteran eusocial insects (ants, some bees and wasps) are highly biodiverse, have an increasing number of species with sequenced genomes, and have databases of ecological and trait data (e.g., Parr et al., 2017; Elsik et al., 2018; Friedman et al., 2020a). Additionally the Hymenoptera present with several independent originations of fully eusocial living, as well as numerous elaborations (such as agriculture, polymorphic workers, symbioses, etc.). Hymenoptera are thus a promising group of species in which to study multiscale ecological relationships using the active inference framework.

## Data Availability Statement

Publicly available datasets were analyzed in this study. This data can be found at: https://github.com/alec-tschantz/ants.

## Author Contributions

DF and AC wrote the first draft, AT and AC designed the simulation, and MJDR and KF made substantial additions to the manuscript. All authors contributed to the article and approved the submitted version.

## Conflict of Interest

The authors declare that the research was conducted in the absence of any commercial or financial relationships that could be construed as a potential conflict of interest. The handling Editor declared a past co-authorship with one of the authors DF.
