# Supplementary material for: Active Inferants: An Active Inference Framework for Ant Colony Behavior
Source: Front Behav Neurosci. 2021 Jun 24;15:647732. doi: 10.3389/fnbeh.2021.647732 (PMC8264549; doi:10.3389/fnbeh.2021.647732)
Supplement: Supplementary file 8 [file Data_Sheet_1.pdf]

Appendix

Variational inference is a method for approximate Bayesian inference and depends on two distributions: the variational distribution and the generative model. The variational distribution is a distribution over all unknown variables (states and policy) in the model and represents the agent's beliefs about the current state of the world. The generative model describes an agent's "model" of the world, and specifies a mapping from hidden states to observations. Variational inference then looks to invert this mapping and recover the mapping from observations to hidden states.

More formally, let x refer to hidden variables, and p refer to a policy (fixed sequence of actions). The variational distribution can then be factorized as follows:

$$Q(x_{0:T}, \pi) = Q(\pi) \prod_{t=0}^{T} Q(x_t) \tag{1}$$

Moreover, the agent's generative model can be factorized as:

$$P(o_{0:T}, x_{0:T}, \pi) = P(\pi) P(x_0) \prod_{t=1}^{T} P(o_t \mid x_t) P(x_t \mid x_{t-1}, \pi) \tag{2}$$

Given these distributions, inference is achieved by optimizing the variational distribution in order to minimize free energy:

$$\begin{aligned} Q^*(x_{0:T}) &= \text{argmin}_Q \, F(Q, o_{0:T}) \\ F(Q, o_{0:T}) &= \mathbb{E}_{Q(x_{0:T})}[\ln Q(x_{0:T}) - \ln P(o_{0:T}, x_{0:T}, \pi)] \end{aligned} \tag{3}$$

In a similar fashion, action selection is achieved by optimizing the variational distribution to minimize expected free energy:

$$\begin{aligned} \pi \sim Q(\pi) &= \sigma\big(-G(\pi)\big) \\ -G(\pi) &= \mathbf{E}_{Q(o_{t:T}, x_{t:T}|\pi)}\big[\ln Q(x_{t:T}|\pi) - \ln P(o_{t:T}, x_{t:T}|\pi)\big] \end{aligned} \tag{4}$$

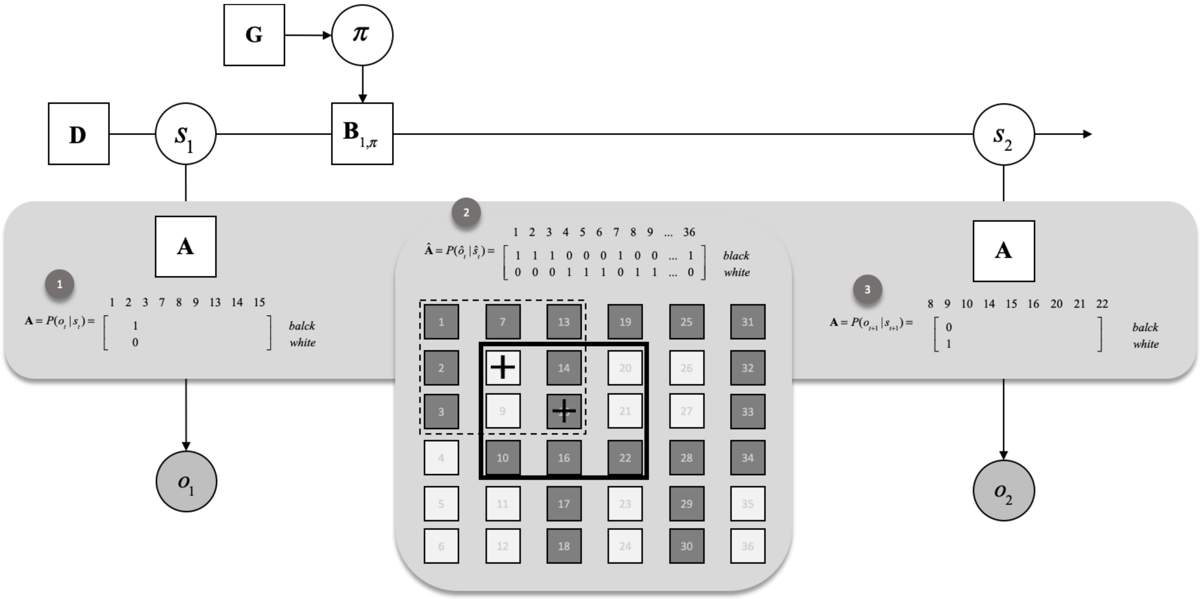

Figure S1. Illustrative example of spatial likelihood remapping. A generative process (A hat) is shown here for 36 locations in a 6 by 6 grid around an agent (center cross) that has a local field of sensory and action accessibility (bounded box around the center cross). Each location, regardless of agent sense or action, has a hidden true state of the world (such as pheromone density in the ant case). Each hidden state is associated with an outcome (visualized as either black or white here, but simulated as a discretized pheromone density scale in the simulation in the main text). (1): the initial likelihood is defined for an agent that would start in location 8. The likelihood of the generative model is specified based on the 8 locations, or hidden states surrounding the current location, as well as the current location (dotted square in panel (2)). (2): Based on the inferred policy (e.g., 8 → 15), we move the agent in the generative process, here, to location 15. (3): Indexing the novel surrounding and current location from the generative process (full lines square in panel (2)), we remap the likelihood that will be used at t+1 to infer the state and the policy.
